# Catalytic Mechanism of Cellulose Degradation by a Cellobiohydrolase, CelS

**DOI:** 10.1371/journal.pone.0012947

**Published:** 2010-10-12

**Authors:** Moumita Saharay, Hong Guo, Jeremy C. Smith

**Affiliations:** 1 University of Tennessee/Oak Ridge National Laboratory Center for Molecular Biophysics, Oak Ridge National Laboratory, Oak Ridge, Tennessee, United States of America; 2 Department of Biochemistry and Cellular and Molecular Biology, University of Tennessee, Knoxville, Tennessee, United States of America; University Paris Diderot-Paris 7, France

## Abstract

The hydrolysis of cellulose is the bottleneck in cellulosic ethanol production. The cellobiohydrolase CelS from *Clostridium thermocellum* catalyzes the hydrolysis of cello-oligosaccharides via inversion of the anomeric carbon. Here, to examine key features of the CelS-catalyzed reaction, QM/MM (SCCDFTB/MM) simulations are performed. The calculated free energy profile for the reaction possesses a 19 kcal/mol barrier. The results confirm the role of active site residue Glu87 as the general acid catalyst in the cleavage reaction and show that Asp255 may act as the general base. A feasible position in the reactant state of the water molecule responsible for nucleophilic attack is identified. Sugar ring distortion as the reaction progresses is quantified. The results provide a computational approach that may complement the experimental design of more efficient enzymes for biofuel production.

## Introduction

The generation of fuels from cellulosic biomass is a promising avenue in renewable energy research [1]–[4]. Cellulose, the most abundant carbohydrate produced by plants, is a linear chain of glucose subunits linked by 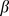-1,4 glycosidic bonds with a repeating unit of a cellobiose disaccharide. Cellulose forms crystalline, insoluble microfibrils in plant cell walls which are recalcitrant to enzymatic hydrolysis. This recalcitrance is the bottle-neck in cellulosic ethanol production [2].

Cellulolytic microorganisms produce a battery of enzymes, called cellulases, exhibiting synergistic activity [5]–[7] in the enzymatic hydrolysis of cellulose to glucose [8]–[10], and are thus of considerable interest in bioenergy research. A particularly biochemically well-characterized cellulase is the cellobiohydrolase *CelS*, an extracellular exoglucanase of bacterial origin that catalyzes the hydrolysis of the glycosidic bond in cellulose. CelS is the major enzymatic component of the *Clostridium thermocellum* cellulosome [11], [12]. The amino-acid sequence of the catalytic domain indicates that *CelS* is a Family 48 enzyme as classified in Refs. [13]–[15]. All Family 48 enzymes are known to liberate cellobiose moieties by a processive mechanism [16]–[18]. Catalytically, these enzymes generally use a single displacement mechanism resulting in inversion of the anomeric configuration [19], [20]. The activity of recombinant CelS on amorphous cellulose has a pH optimum of 5–6 at 70
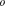
C [21].

**Figure 1:**
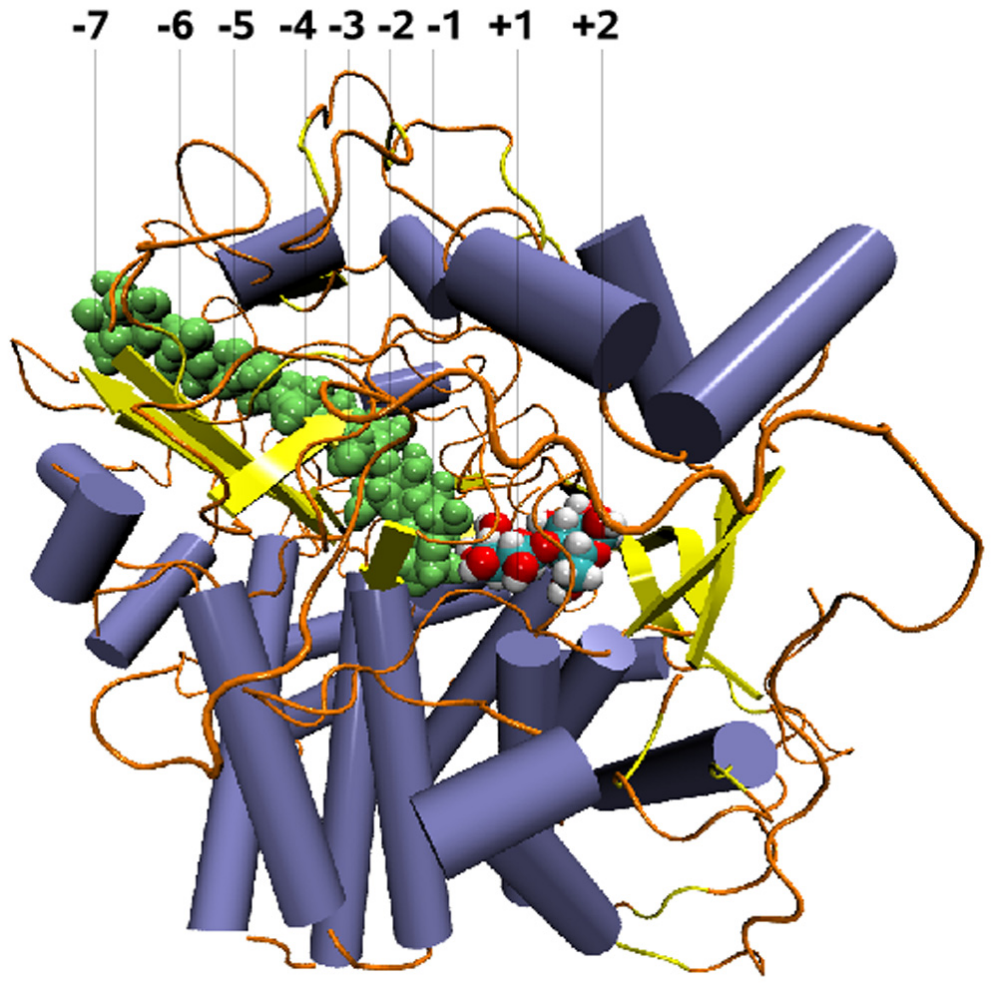
Model of cellobiohydrolase CelS structure in complex with celloheptaose (green), and cellobiose, colored in white (hydrogen), red (oxygen), and cyan (carbon). The sugar unit at subsite −1 was modelled manually. The substrate, celloheptaose, spans between subsites −1 to −7 in the substrate-binding tunnel, and cellobiose is bound in the cleft region between subsites +1 and +2. This figure was made using the VMD software [66].

**Figure 2:**
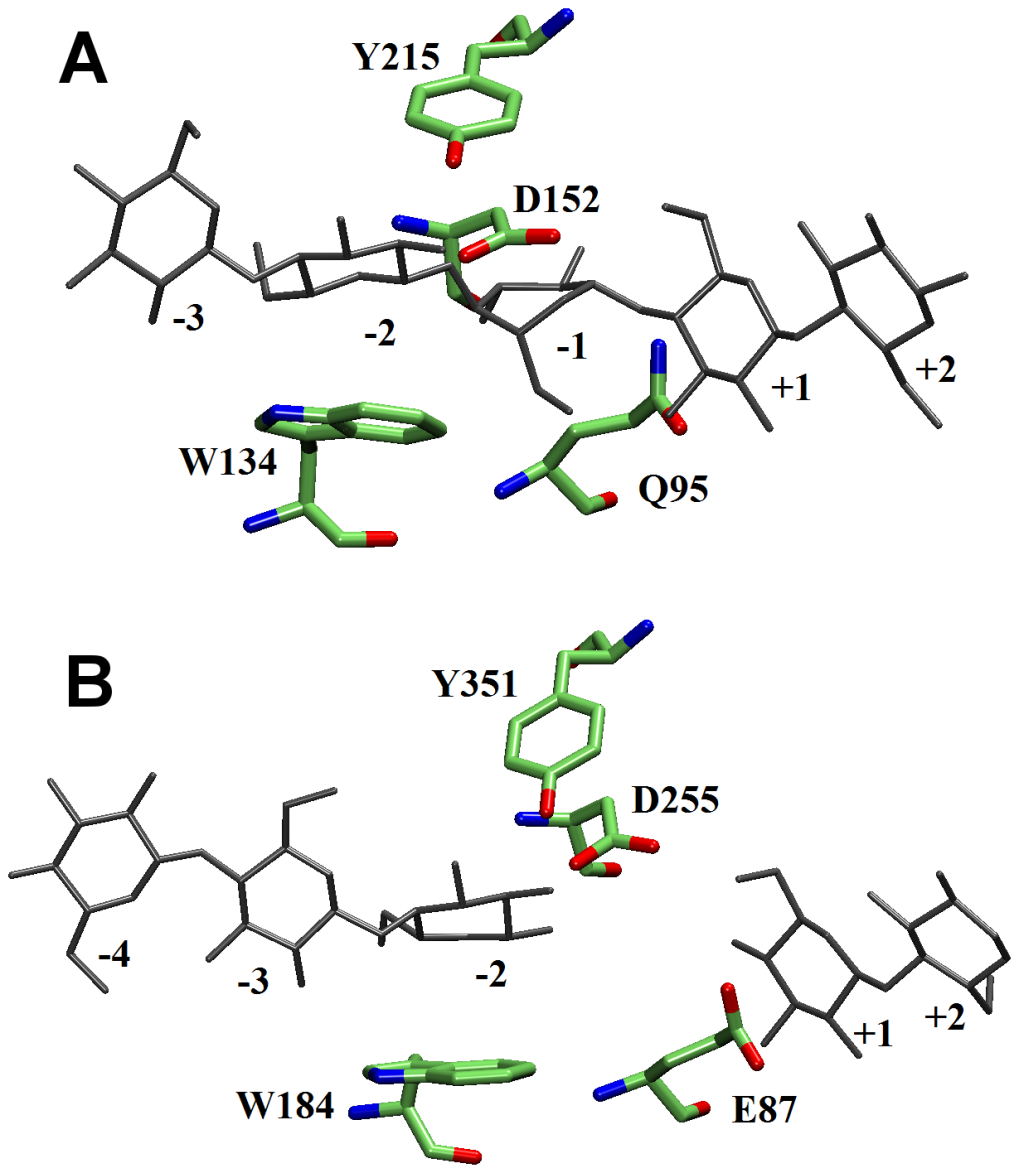
Structural comparison of active sites of (A) family 8 CelA and (B) family 48 CelS. Substrate is shown in Gray.

The CelS crystallographic structure [21] is shown with labeled sugar-binding subsites in Fig 1. Two residues in the active site are believed to play key roles in the catalysis : one contributes general base assistance to the attack of the nucleophilic water, and the other acts as a general acid in the cleavage of the glycosidic bond [22]. Determination of the participating catalytic residues is an essential prerequisite to elucidating the detailed reaction mechanism. Structural comparison with another Family 48 enzyme, CelF, which has been crystallized in the product state, suggests that Glu87 is likely to be the general acid in CelS [23]. Indeed, in CelS, Glu87 is in proximity to the active site and makes favorable hydrogen-bonding interactions with the O4 atom of the sugar unit at subsite +1 [21], although confirmation of the functional role of Glu87 is still lacking. In contrast, the general base has not been unequivocally identified, due in part to the absence of a sugar unit at sugar binding subsite 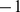 in the CelS crystal structure. The structural comparison (Fig 2) with a family 8 enzyme, CelA [24], which also has similar structure as CelS, suggests that one candidate residue could be Asp255 [21], [25]. However, inspection of the crystal structure with a sugar unit modeled at subsite −1 has led to the suggestion that Asp255 is more likely to stabilize the sugar ring boat conformation (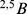) at this subsite rather than being the base catalyst [21]. As an alternative to Asp255, Tyr351 might participate in the reaction mechanism, although a direct catalytic role might be precluded by its high pKa value in an acidic environment. Another important reactant is the nucleophilic water molecule, which donates a hydroxyl ion to the anomeric carbon atom at subsite −1, and has been predicted to stabilize the oxocarbenium-type transition state of the central sugar ring at subsite −1 after glycosidic bond cleavage [24]. A difficulty in experimental investigations is the elucidation of the position of this water molecule as it exists only in the reactant state.

Experimental studies on cellulases suggest that the binding of a glucosyl unit at subsite 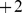 in the reactant state induces strain on the glucosyl unit at subsite −1 involving ring distortion [26]–[28]. This conformational strain could facilitate the reaction by producing a ring structure in the reactant state that is close to the transition state [29]–[31].

Given the existence of appropriate biochemical and high-resolution crystallographic experiments, computer simulation studies can be performed to probe the roles of the catalytic residues. A recent QM/MM study [32] on the mechanism of cellulose hydrolysis by inverting GH8 enzymes indicated that Asp278 acts as the general base in a mechanism with a calculated activation free energy of 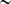
24 kcal/mol. Classical molecular dynamics simulations on another inverting enzyme Cel6A [33] have been performed to examine the catalytic residues at the active site and have identified Asp221 as the catalytic acid and Asp175 as the probable proton acceptor. In another related study, a quantum mechanical/molecular mechanical (QM/MM) free energy calculation [34] of the inverting reaction mechanism in human O-GlcNAcase involving substrate-assisted catalysis in the hydrolysis of N-acetyl-glucosamine hemiacetal found a barrier height of 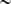
11 kcal/mol. Along the QM/MM trajectory, the ring conformation at the reactant state was observed to be a distorted 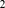
S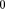. Ring distortion conformational energetics were also characterized in *ab initio* metadynamics simulations [35] performed on gas-phase 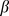-D-glucopyranose. Nine free-energy minima were observed with 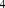
C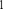 as the most stable conformation. QM/MM calculations on the Michaelis complex [36] of Bacillus 1,3-1,4-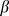-Glucanase showed that this simulation methodology comprise useful information on the transition states in glycosyl hydrolases.

In the present article, QM/MM calculations are reported on CelS (PDB ID code 1L2A) with the goal of identifying a plausible reaction mechanism and the identity of the general base. The results suggest that Asp255 is the most probable base catalyst. Further, the position of the nucleophilic water molecule in the reactant state is derived together with the roles of this water molecule and the catalytic residues (Glu87 and Asp255) in the reaction mechanism. Structures of different putative catalytic states are obtained as a function of the glycosidic bond length. We examine also how the pyranose ring at the catalytic center (at subsite −1) may undergo conformational changes to facilitate the reaction.

## Results and Discussion

### Determination of the Reaction Coordinate

Initially, reaction path calculations were performed with Tyr351 as the base and Glu87 as the acid catalyst. A variety of putative reaction coordinates were examined, but it was found to be impossible to locate a stable reactant structure in which the nucleophilic water molecule is at a reasonable distance from the anomeric carbon at subsite −1 and also makes a strong hydrogen bonding interaction with the base catalyst [24]. Instead, this water molecule was situated at least 3.8Å away from the hydroxyl group of Tyr351. Thus, it is unlikely that Tyr351 is the base catalyst. In contrast, using Asp255 it was possible to obtain both a stable reactant state and a physically and energetically reasonable reaction pathway, consistent, therefore, with Asp255 being the base catalyst. Consequently, the results of the calculations with Asp255 and Glu87 as the acid and base catalysts are presented in the following sections.

**Figure 3:**
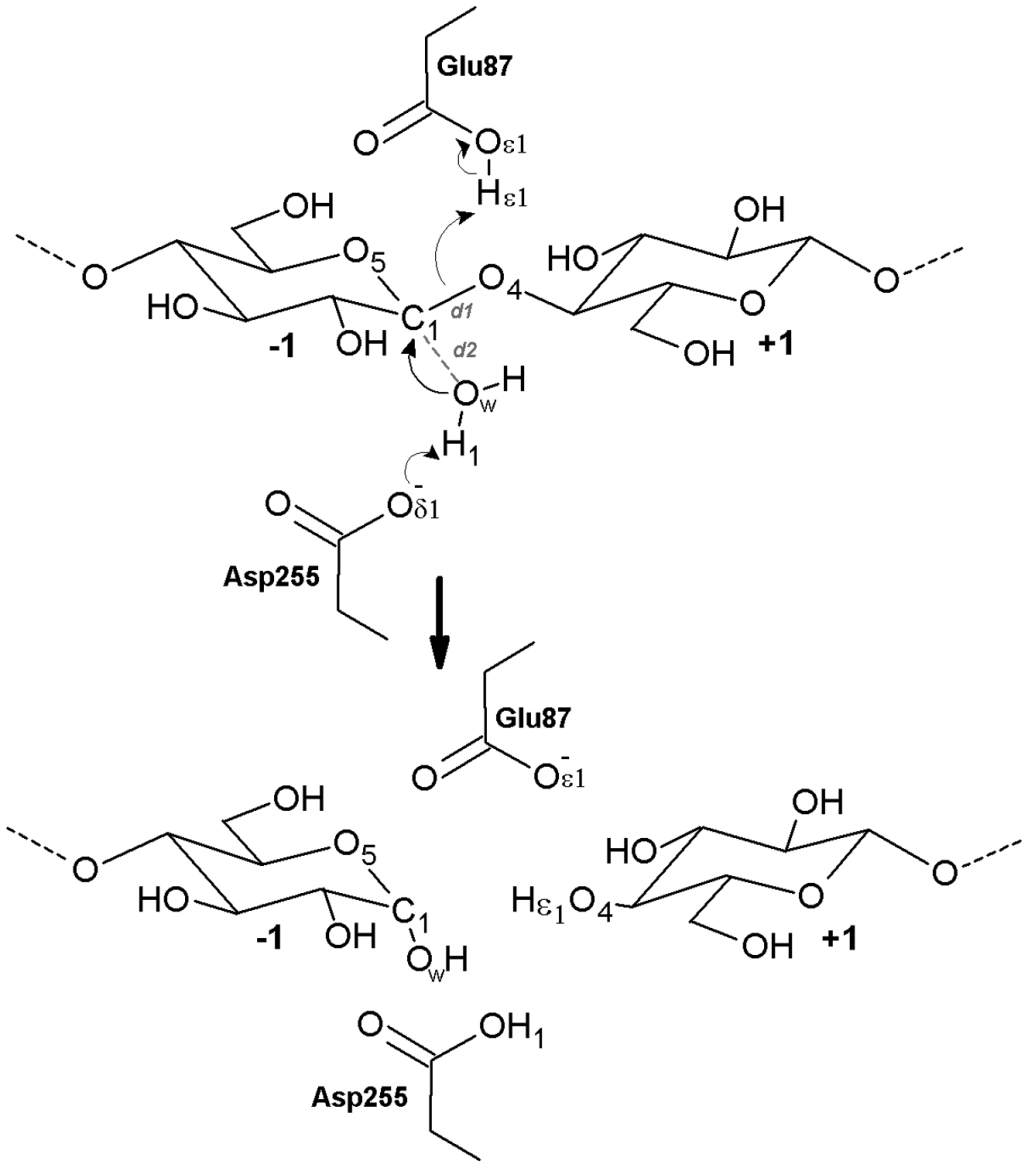
Schematic representation of inverting reaction mechanism in CelS for hydrolysis of glycosidic bond C1-O4. The catalytic residues Glu87, Asp255, and nucleophilic water molecule (W1) are shown. Anomeric carbon atom at subsite −1 and leaving group oxygen atom at subsite +1 are C1 and O4, respectively. The thin arrows represent electron transfer between atoms. Distances between atoms are shown in red.

As is shown in Fig 3, several covalent and non-covalent interactions are involved in the bond breaking and making processes during the enzyme catalysis. A detailed description of the enzyme-catalyzed reaction would then require mutiple reaction coordinates extremely difficult to determine and fully sample. Therefore, we adopted an alternative approach.

- In an initial set of calculations, a one-dimensional free energy profile was generated with the distance between C1 and O4 (d1) as the reaction coordinate (RC). Although a spontaneous proton transfer from Glu87 to the leaving group oxygen (O4) upon C1-O4 bond cleavage was found, to accompany this change there was no nucleophilic attack by the water molecule.
- In order to assist the nucleophilic attack, in a second set of calculations the reaction coordinate (RC) was specified as the difference in distances (d1–d2) between C1 and O4 (d1) and between the water oxygen (O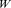) and C1 (d2). The free energy difference between the reactant (RC = −1 Å) and the product (RC = 1.5 Å) states was 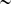
50 kcal/mol. In comparison, the observed thermodynamic activation parameter (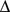
H) for an inverting enzyme [32] is 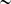
12 kcal/mol which is much lower than the calculated energy difference between the substrate and product in CelS. However, despite the large change in energy for the transition from the reactant to the product state along this reaction pathway, the geometric structure of the product was reasonably stable.
- Thirdly, two reaction coordinates were chosen: RC1, the distance between C1 and O4, and RC2 (RC2 = d1+d2+d3), a linear combination of d1 (proton transfer from Glu87 to the leaving group), d2 (the distance between the nucleophilic water oxygen (O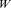) and C1), and d3 (proton transfer from W1 to Asp255, *i.e.*, the difference in distances between O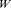-H1 of W1 and O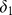 of Asp255-H1). The reactant and product states were represented by (RC1 = 1.2, RC2 = 3.5 Å) and (RC1 = 3.2, RC2 = 0.0 Å), respectively. The two-dimensional free-energy profile again for this path shows a high energy barrier (40 kcal/mol).
- The above considerations indicate that, due to the complexity of the reaction mechanism (a single displacement inverting mechanism), in which the reaction rate depends on the protonation state of the base catalyst [37], it is very difficult to generate the reaction-pathway on a low dimensional free-energy landscape. Finally, therefore, four coordinates (RCs) were required for satisfactory description of the hydrolysis of the glycosidic bond (Fig 3). The choice of a relatively high number (more than two) of reaction coordinates to represent this kind of concerted reaction has also been found necessary in previous *ab initio* [38] and QM/MM [39] calculations. The coordinates used here are, RC1: proton transfer from Glu87 to the leaving group, *i.e.*, the difference in distances (d3–d4) between H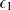-O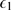 (d3) on Glu87 and the leaving group glycosidic oxygen (O4)-H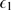 (d4), RC2: glycosidic bond cleavage, *i.e.*, the distance between the anomeric carbon (C1) and O4, RC3: the distance between the nucleophilic water oxygen (O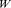) and C1, and RC4: proton transfer from W1 to Asp255, *i.e.*, the difference in distances between O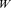-H1 of W1 and O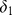 of Asp255-H1. The reaction coordinates are similar to that used in the very recent QM/MM analysis on GH8 endoglucanases [32].

The following simulation protocol was employed to calculate the reaction mechanism. The reaction coordinate which quantifies the glycosidic bond length, RC2, was used as a progress variable. An efficient iterative optimization procedure was used to obtain the optimized structures and minimum energy pathways along the reaction coordinates [40]. The minimum-energy pathway along RC2 subject to constraints on the other coordinates (RC1, RC3, RC4) traces out a pseudo-one dimensional stripe through a four-dimensional space. In the first step, the structure of the QM/MM system in each RC2 window was optimized with constrained RC1, RC3, and RC4 coordinates. An energetically reasonable path was found to be that in which the four RCs were incremented simultaneously.

Subsequently, umbrella sampling calculations [41] were performed along the reaction pathway in windows starting from the corresponding optimized structures. The Weighted Histogram Analysis Method (WHAM) [42] was used to unbias the umbrella-sampling results along the RC2 reaction coordinate, *i.e.* along the glycosidic bond cleavage. Harmonic restraints with force constants of 100 kcal/mol/Å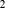 for proton transfer (RC1, RC4) and 500 kcal/mol/Å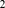 for covalent bond breaking/formation (RC2, RC3) were used to guide the inverting reaction mechanism. 42 windows were computed, each with 10 ps of equilibration and 10 ps of production run.

### MD Simulation of Product State

As mentioned in Methods, the hydrolysis reaction was derived in the reverse direction *i.e.*, from the product (cleaved) state to the reactant. Here, we first examined the structural properties of the starting (product) state subjected to 7ns classical MD simulation. The time evolution of the enzyme backbone RMSD (root mean square deviation) in the product state compared to the lowest-energy conformation was calculated. The RMSD increases during the initial 2 ns of MD trajectory then stabilizes at 1.5Å indicating that the product state structure of the enzyme is stable. The fluctuation amplititude of the active site water molecule is 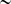
1.5Å. Hence, during the timescale of the present simulations there is no exchange with the bulk.

In the equilibrated system, the carboxylate oxygen atom of Glu87 forms a strong, stable hydrogen bond with the OH-4 atom of the cellobiose fragment at subsite +1. Tyr351, one of the putative base catalysts, hydrogen bonds with OH-3 at subsite −1 and is positioned 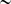
5.5Å away from the anomeric center (C1 of subsite −1) whereas, the distance between C1 and Asp255 is 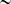
5.3Å. The carboxylate oxygen atom (O
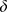
1) of Asp255 is oriented towards the anomeric center and is 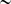
4.5Å away from the OH-1 at subsite −1. In contrast, OH of Tyr351 is further away (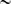
5.5Å) from the OH-1. The organization of the OH-2, and OH-1 atoms at subsite −1 near Tyr351 might hinder nucleophilic attack at the anomeric center in the reactant state whereas, Asp255 is in more favourable position. As described in the “Methods”, attempts to find a low-energy reaction path with Tyr351 as the general base failed. Consequently, Asp255 is likely to be a better candidate for the base catalyst than Tyr351.

### Energy Profile

The present postulated inversion reaction mechanism of enzymatic hydrolysis in CelS involves a proton transfer from the acid catalyst (Glu87) to the leaving group O4 and a nucleophilic attack by a water molecule at the anomeric center (C1) assisted by the base catalyst (Asp255). Minimum-energy pathways were obtained for both the forward and reverse reactions, and the enthalpy difference between the transition and reactant states found to be 32 kcal/mol.

**Figure 4:**
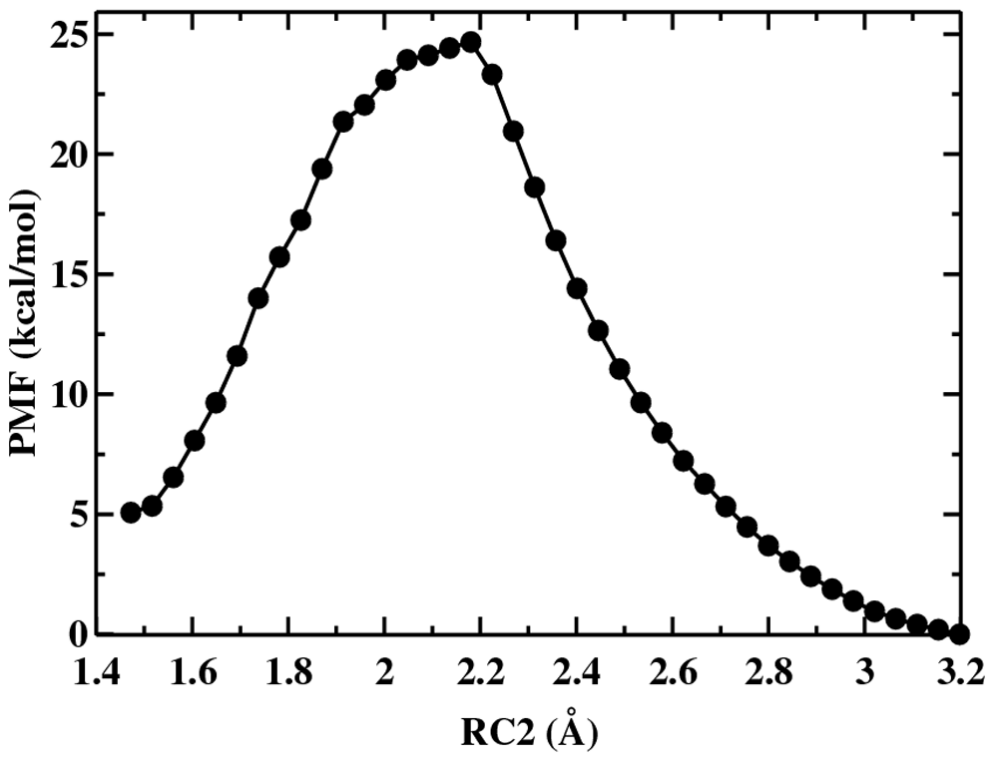
Potential of mean force for hydrolysis reaction.

The free energy profile (Fig 4) for the hydrolysis mechanism was obtained by umbrella sampling calculation along the reaction coordinate corresponding to the minimum energy pathway. The reactant state is at RC2 = 1.5 Å at which point a contiguous cellulose chain is bound in the substrate binding tunnel. The free-energy barrier is 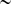
19 kcal/mol. Therefore, there is a significant reduction in barrier height (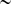
13 kcal/mol) relative to the potential energy, a part of which may be attributed to the entropic contribution as well as the relaxation of the system during the MD simulations.

The reaction proceeds through a transition state at RC2 = 2.17 Å. Recalculation of the free energy barrier starting from a slightly different reactant state configuration led to a barrier of 21 kcal/mol. This difference of 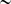
2 kcal/mol near the transition state arises from relatively poor sampling around the unstable transition state configurations. However, the two free-energy profiles resemble each other closely during the passage from the transition state to the product. The standard deviation between the two free energy profiles is 0.94 kcal/mol. Finally, a stable product state is formed at 3.2 Å. The change in free energy between product and reactant states in two systems are 5 and 4 kcal/mol for the two calculations, respectively.

**Figure 5:**
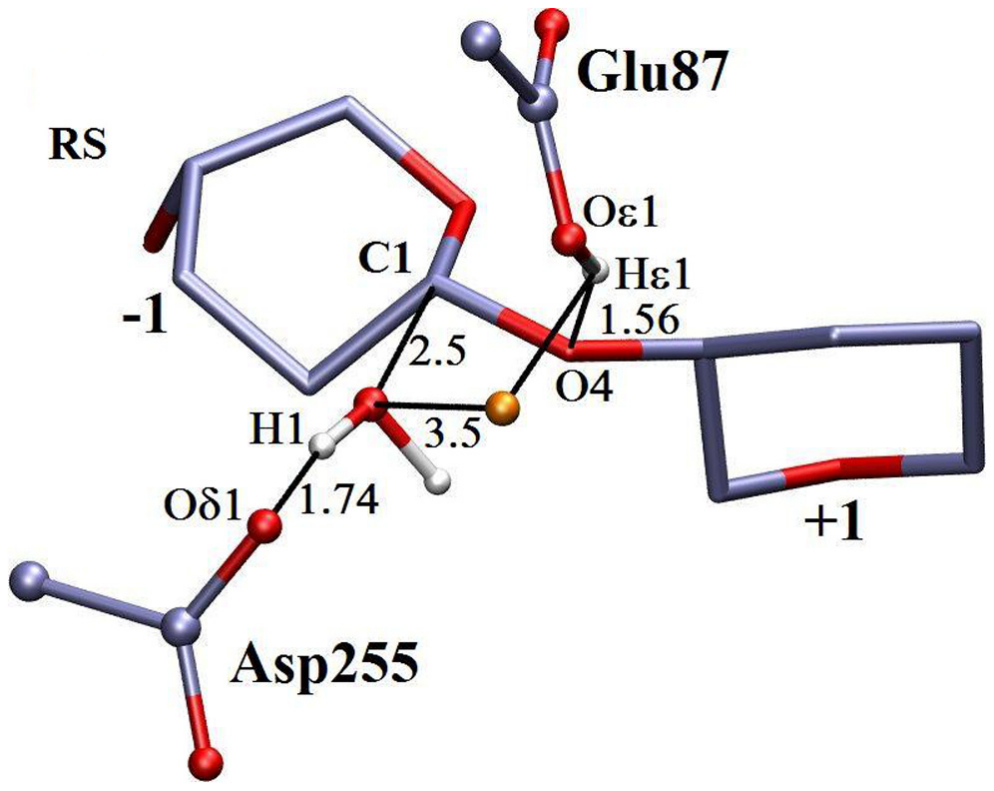
Snapshot of reactant state from QM/MM molecular dynamics free energy trajectories. Only the active site residues, glucosyl units −1 and +1, acid catalyst Glu87, base catalyst Asp255, nucleophilic water, and a second water molecule shown in gold (omitted Hydrogen atoms for clarity), are shown here. All backbone atoms of Glu87 and Asp255 are omitted for clarity. Important distances are shown by black lines.

**Figure 6:**
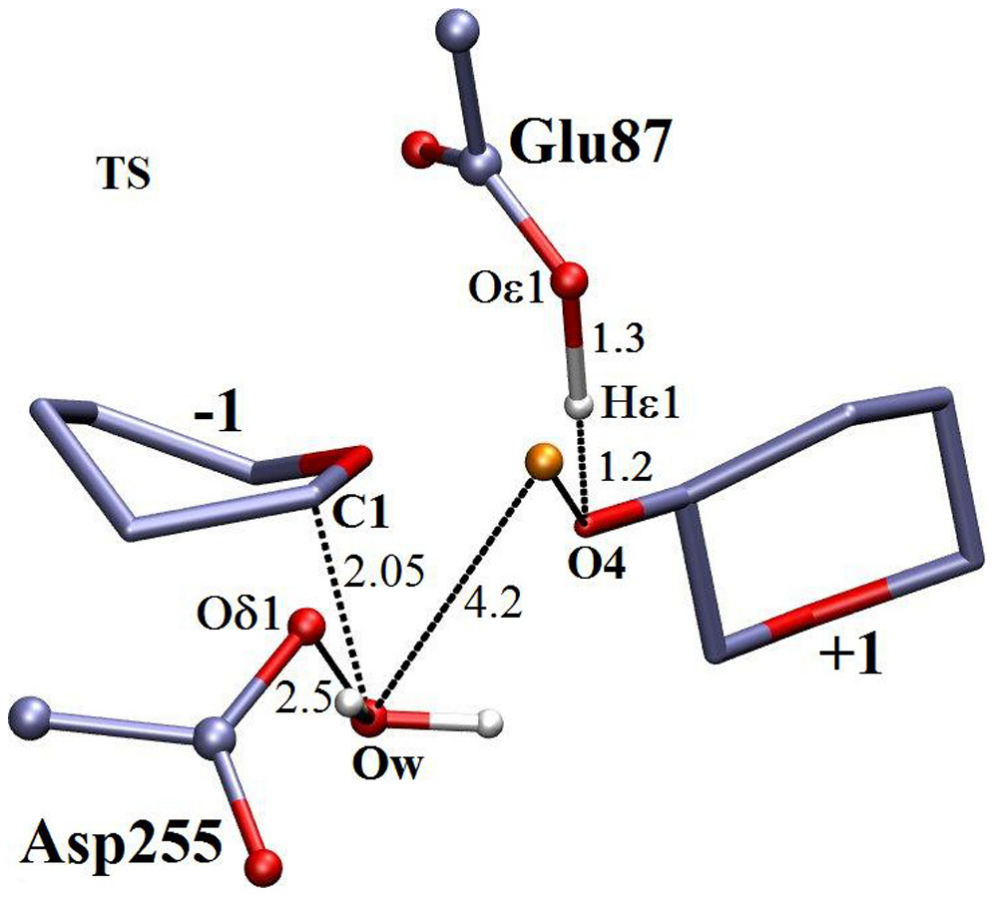
Snapshot of transition state from QM/MM molecular dynamics free energy trajectories. Other figure specifications are similar to Fig 5.

**Figure 7:**
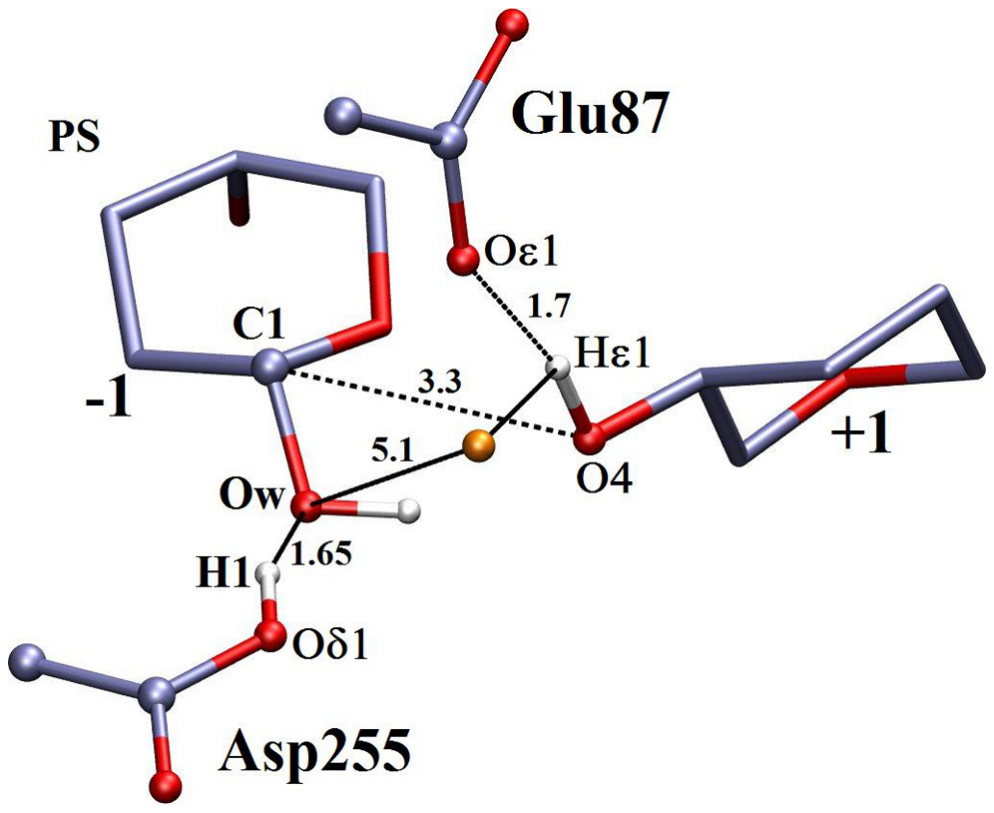
Snapshot of product state from QM/MM molecular dynamics free energy trajectories. Other figure specifications are similar to Fig 5.

Three snapshots obtained from the QM/MM simulations, representing the reactant state (RS), the structure near transition state (TS), and the product state (PS) together with important geometric parameters are shown in Fig 5, 6, 7.

### Reactant State

The crystallographically-derived model structure of CelS in complex with two oligosaccharides [21] provides the coordinates of the product state, *i.e.* after glycosidic bond cleavage. The product state does not contain the water molecule that plays the role of the nucleophilic attack of the anomeric carbon in the reactant state. However, the reverse reaction mechanism (*i.e.*, starting from the product state) was calculated and allows the position of this water molecule in the reactant state to be derived. Indeed, Fig 5 clearly shows the formation of a water molecule (W1) near the anomeric center. This water molecule makes a hydrogen-bonding (distance between H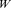 and O
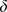
1 of Asp255 is 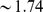
Å) interaction with the base catalyst, Asp255. Glu87 becomes protonated and forms a hydrogen bond with the glycosidic oxygen (O4).

In the crystal structure of liganded CelA [24], a Family 8 inverting enzyme with Glu95 as the proton donor and Asp278 as base catalyst, a potentially nucleophilic water molecule was seen in the electron density map, making a strong hydrogen bonding interaction with the base catalyst at a distance of 2.8 Å from C1 at subsite −1. The present modeled reactant state in CelS exhibits a geometry similar to that of CelA, apart from the fact that the nucleophilic water molecule in CelA is strongly hydrogen bonded with two active site residues, Tyr215 and Asp278 (the general base), whereas, in contrast, Tyr351 in CelS (equivalent to Tyr215 in CelA) is positioned relatively far away (6.4 Å) from the nucleophilic water molecule, indicating that it makes no significant contribution to the reaction mechanism.

In the present CelS reactant state, a second water molecule (W2) (Fig 5) forms hydrogen bonds with both W1 and Glu87. Thus, a chain of two water molecules (W1, W2) hydrogen bonds to the hydroxyl of Glu87 and carboxylate of Asp255. Throughout the QM/MM simulation, the sidechain of Glu87 remained hydrogen bonded with W2, in a geometry similar to that observed in the crystal structure of another inverting enzyme, the cellobiohydrolase Cel6A [33]. This water molecule was not included in the QM region. The hydrogen bonding scenario implies that W2 may help stabilize the product state, although, the exact role of this molecule is not completely understood here.

### Transition State

The reaction proceeds through a highly dissociative transition state with increasing charge formation at the anomeric center and the formation of a partial double bond between the C1 and O5 atoms leading to an oxocarbenium ion-like structure [36]. In the transition state (Fig 6), distortion is found in the pyranose ring at subsite −1 with the bond length C1-C2 (1.58 Å) increased by 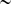
0.04 Å and C1-O5 (1.3 Å) decreased by 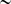
0.16 Å. The anomeric center (C1) assumes a position approximately 2.0 Å away from W1 with the leaving group (O4 at subsite +1) at a distance of 2.3 Å. The leaving group is partially bonded to the catalytic acid Glu87 as the proton (H
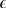
1) from Glu87 is shared between these two groups with the distances O
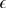
1-H
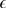
1 and H
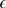
1-leaving-group-O4 being 1.3 and 1.2 Å, respectively. W2 makes a hydrogen bonding interaction with Glu87, which may play an important role in stabilizing the TS structure. The distances H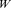-O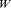 and H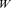-O
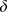
1 of Asp255 are 1.1 and 1.3 Å, respectively, implying that the proton transfer from W1 to Asp255 has not taken place at this stage. Thus, in the proposed mechanism, a proton transfer from the acid catalyst Glu87 to the glycosidic oxygen is followed by the nucleophilic attack at the anomeric carbon (C1). This mechanism is similar to that calculated using QM/MM analysis for GH8 inverting enzymes [32].

### Product State

In the product state (Fig 7), the C1 atom of the sugar ring at subsite −1 forms a covalent bond (1.43Å) with the hydroxyl group of the dissociated water molecule and Asp255 is protonated, where the H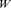-O
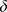
1 distance is 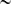
1.0Å. After the hydrolysis, the product (cellobiose) is slightly displaced from the active site with a C1-O4 distance of 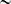
3.3 Å.

### Sugar Ring Distortion

Upon binding to the enzyme, the sugar ring at subsite −1 undergoes a conformational change [36] from an undistorted, 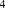
C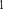 chair structure to a distorted, 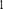
S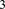 skew-boat structure. This arises from (a) the charge increment at the anomeric carbon atom (C1), (b) the increment in the distance between C1 and O4 of the leaving group, and (c) a decrease in the intra-ring O5-C1 distance. The carbohydrate interacts with the protein mainly *via* hydrogen bonding and stacking interactions involving the aromatic side chains, and these interactions produce continuous torsional strain on the substrate, resulting in substantial conformational change at subsite −1 that may weaken the scissile glycosidic linkage. The X-ray crystallographic analysis of the retaining reaction mechanism in Cel5A [43] in various stable states shows that the bound substrate undergoes substantial distortion from favoured 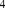
C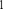 conformation to distorted 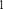
S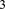 skew-boat in the Michaelis complex and regains the undistorted 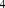
C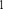 conformation in both the glycosyl-enzyme intermediate and the product state after hydrolysis. In contrast, experimental evidence [24] on the inverting enzyme, Cel8A, indicates a distorted 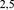
B conformation of the glucosyl unit at subsite −1 upon binding to the enzyme.

### Sugar Ring in Reactant State

**Figure 8:**
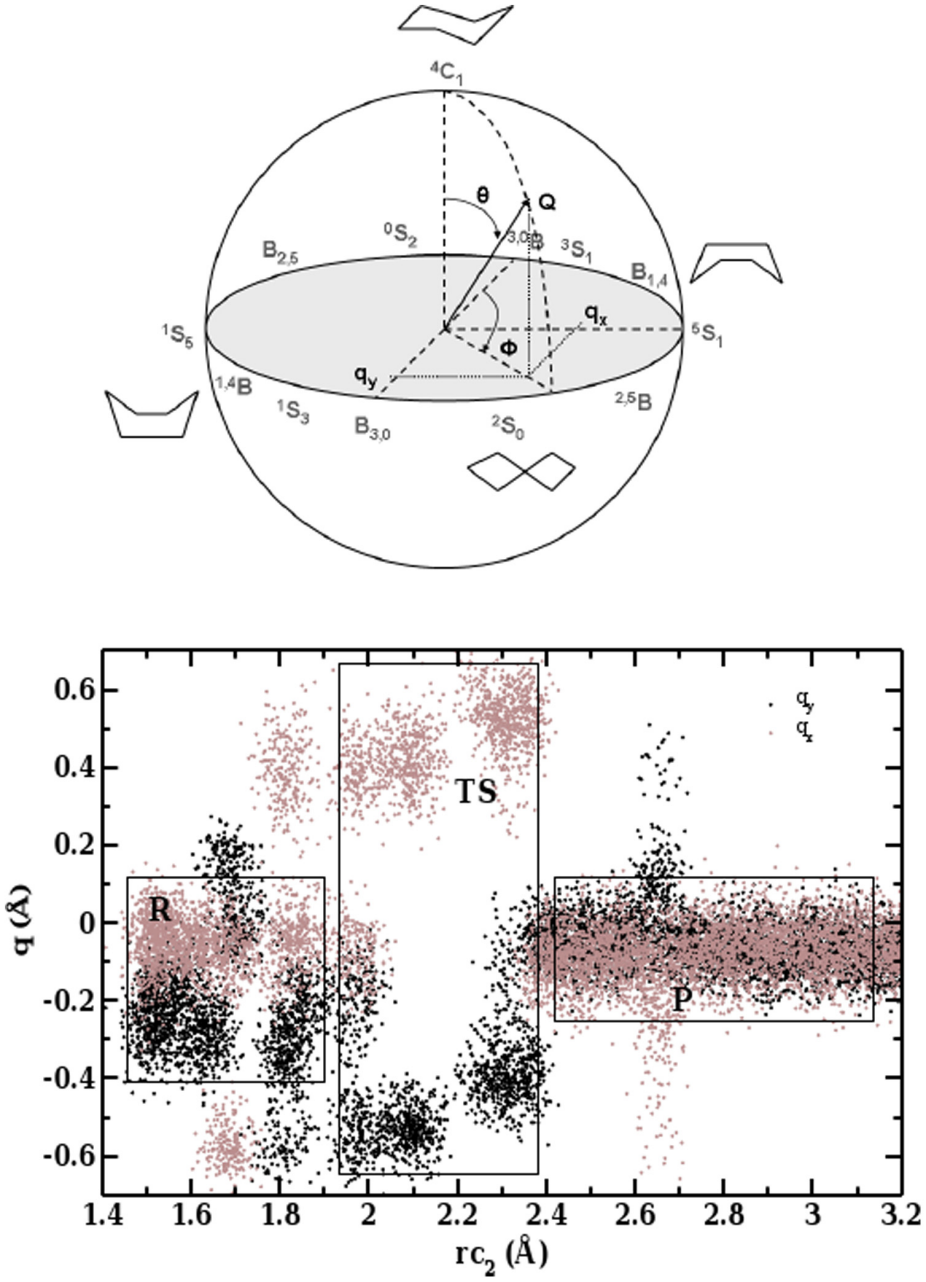
Upper panel: Puckering coordinates (Q,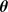,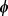) for six membered ring. Lower panel: Projection of puckering coordinates (q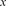, and q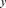) sampled by molecular dynamics trajectories along the reaction coordinate rc2. q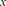 and q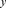 values are shown in gray and black, respectively. Regions corresponding to reactant (R), around transition state (TS), and product (R) are highlighted by boxes.

The extent of the distortion from the 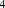
C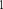 conformation can be quantified by a set of ring puckering coordinates [44]. All accessible conformers of a sugar ring due to ring distortion can be represented by Stoddarts pseudopotential itinerary [35] in terms of q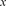 and q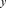 values. Here, the puckering coordinates (Q,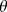,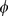) (Fig 8) and the two-dimensional projections (q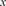 = Q sin 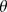 sin 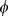, q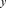 = Q sin 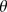 cos 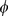) for the hexopyranose ring conformations were calculated as described in Ref. [35].

Fig 8 displays the change in sugar ring conformation in terms of q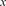 and q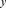 at the major catalytic center (subsite −1) along the reaction coordinate, RC2. The reactant state is associated with 1.5 Å
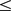
RC2
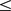
1.8 Å, and assumes predominantly a distorted 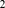
S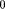 conformation. The angle between C1-O4 and the plane of the sugar ring at subsite −1 is around 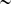
85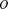 *i.e.* the leaving group is pseudo-axial. This strained geometry, with an axial position of the glycosidic bond, preactivates the substrate for glycosidic hydrolysis [45]. Car-Parrinello molecular dynamics simulations on the gas phase 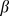-D-glucopyranose ring have shown that the conformers between 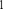
S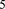 and 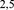
B are the frequently observed structures of the substrate and that the free-energy difference between the undistorted 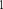
C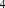 and 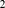
S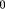 conformers is 3.0 kcal/mol [35]. This can be compared with the present simulation result in which the free-energy difference between the product (in the 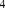
C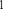 conformation) and reactant (in the 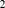
S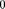 conformation) states is 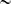
5 kcal/mol.

### Sugar Ring at Transition State

**Figure 9:**
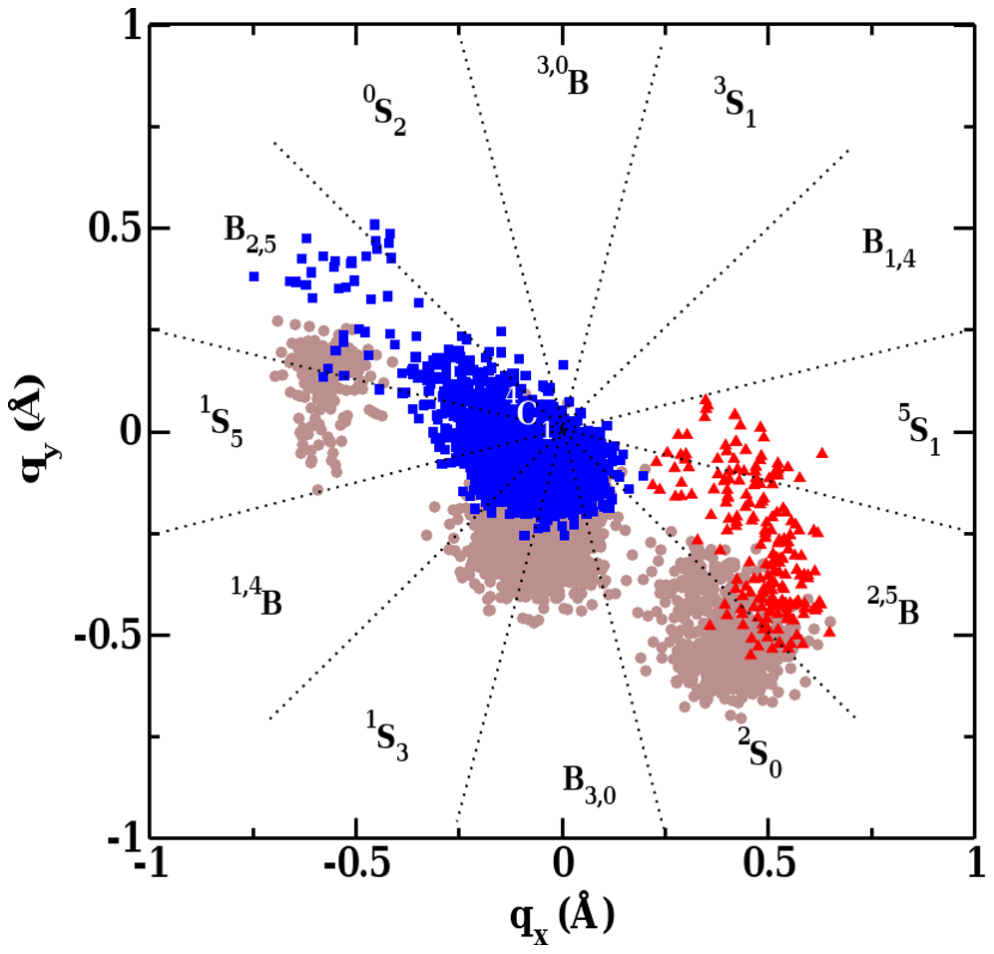
Stoddart diagram showing ring conformations at subsite −1 with respect to q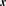 and q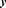. Conformations for reactant (brown circle), transition (red triangle), and product (blue sqare) states are shown. Different regions are separated by dashed black lines. The central region represents undistorted and stable 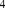
C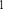 conformation.

As reaction proceeds, a sharp change in q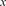 and q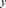 values is found near the transition state (for 2.0 Å
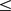
RC2
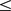
2.3 Å). The distribution of the associated conformers in the Stoddart diagram (Fig 9) indicates that the sugar ring acquires mostly the unstable 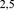
B conformations in the transition state. *Ab initio* calculations on Cellobiohydrolase Cel6A [33] have shown a similar geometry of the high energy and unstable oxocarbenium-type transition state in a 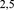
B boat conformation.

During catalysis, the two important internal structural parameters responsible for the conformational change are the C1-O5 and C1-O4 bond lengths. The QM/MM study on the Michaelis complex of 1,3-1,4-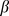-Glucanase [36] revealed that change in conformation at the transition state relative to the reactant state is due mainly to an increase in C1-O1 and a decrease in the C1-O5 bond length by the same amount. These changes in the pyranose ring bring the C5, O5, C1, and C2 atoms into a plane leading to the formation of a partial double bond between C1 and O5 in the 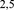
B conformation [46], [47] that facilitates the enzymatic hydrolysis by the inverting mechanism. Correspondingly, in the present calculations, a reduction of 0.15Å in C1-O5 bond length was observed accompanying the transformation from the 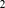
S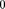 to the 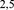
B conformation.

### Sugar Ring at Product State

On the passage from the transition state to the product state, the C1-O5 distance increases with the increasing separation between the substrate and the leaving group. This bond length relaxes to 1.46 Å at the end of the reaction. Both q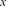 and q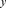 values oscillate around zero in the region 2.4 Å
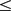
RC2
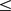
3.2 Å (Fig 8). Finally, in the product state the Stoddart diagram (Fig 9) shows a significantly populated region corresponding to the 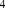
C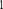 relaxed conformation.

### Conclusions

To investigate the enzymatic reaction mechanism of CelS in cellulose degradation, quantum mechanical-molecular mechanical (QM/MM) simulations of CelS in complex with oligosaccharides have been performed. This Family 48, processive enzyme liberates cellobiose units from the reducing end of cellulose by hydrolysis of the glycosidic bond following an inverting reaction mechanism.

The acid catalysis of the glycosidic bond requires two residues, a proton donor and a base. Based on the structural comparison of CelS complex with other family 48 enzymes, the active-site residue Glu87 has been identified as the proton donor and two residues, Tyr351 and Asp255, were proposed to be the possible candidates for the general base.

The derivation of a structural model of the reactant state of the enzyme-ligand complex, in which a single cellulose chain binds between subsites +2 and subsite −7, was required. This reactant state structure was obtained from the reverse reaction mechanism, in which the nucleophile (water molecule) was one of the reaction products. Several PMF calculations were performed along the reaction pathways selecting either Asp255 or Tyr351 as the catalytic base, and the resulting structures and energetics suggest that Asp255 is the more likely base candidate. Asp255 forms a hydrogen bond with the nucleophilic water and OH2 of the carbohydrate at subsite −1 in the reactant and product states, respectively.

The complexity of the CelS hydrolysis mechanism, in which many degrees of freedom participate chemically, precludes an exhaustive search of the reaction space. However, the calculated activation free energy in the present SCC-DFTB/MM simulation in CelS is 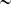
19 kcal/mol, which is in the expected range for the inverting enzymes [32].

The reaction proceeds through an oxocarbenium type transition state in which the glucosyl unit at subsite −1 transforms from the 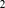
S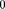 conformation before hydrolysis to the 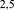
B conformation. In the product state, the cellobiose moiety is slightly pushed to the open cleft region and the sugar ring at subsite −1 regains the undistorted 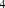
C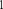 conformation. Detailed examination of the geometry of the anomeric carbon showed that this changes from sp3 at the reactant state to sp2 at the transition state and regains an sp3 configuration in the product state.

The present work identifies an energetically reasonable reaction pathway for cellulose hydrolysis by a cellulase, CelS. The calculations represent progress towards a quantitative understanding of how cellulases stabilize the transition state for cellulose hydrolysis. The application of QM/MM to the cellulases other than CelS is in progress and should lead to interesting comparative studies. Many factors other than the chemical reaction step also contribute to biomass recalcitrance to hydrolysis, such as lignocellulose structure and the mechanical access of the enzymes to the cellulose strand. Computational and experimental investigations of many of these critical processes are presently being pursued with vigor and together with chemical mechanistic work such as the present, will help provide a basis for rational enzyme and biomass design.

## Materials and Methods

### Construction of reactant state structures

A model of the reactant state of CelS was developed using the following procedure. The initial coordinates of the catalytic domain of CelS (PDB ID code 1L2A) were taken from the X-ray crystal structures (Fig 1) in complex with the cellohexaose and cellobiose, solved at 2.5Å (PDB ID code 1L2A) and 2.4Å (PDB ID code 1L1Y) resolutions, respectively [21]. These structures were solved separately and subsite −1 was unoccupied in both. In Ref.[21], a model of CelS in the product state was constructed by filling the open cleft (subsites +1, +2) of the CelS-cellohexaose complex with cellobiose from the CelS-product complex and these coordinates were used for the product state in the present work.

Starting from the crystallographic product state model, trial reactant state structures were generated using three different methods, (i) by joining the cellobiose moiety with the substrate (celloheptaose) to form the C1-O4 glycosidic bond between subsites −1 and +1, (ii) by replacing the oligosaccharides of CelS by a continuous chain of nine glucosyl units in hemithiocellooligosaccharides from the crystallographic structure of the active-site tunnel of mutant enzyme (E55Q) Cel48F, (iii) by forming the C1-O4 glycosidic bond between subsites −1 and +1 via the reverse reaction mechanism.

In the trial structure from method (i), the glucosyl unit at subsite −1 was found to deform to a 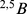 boat conformation. Tyr351 was located about 5.3Å away from the C1 at subsite −1 and Glu87 and Asp255 made hydrogen bonding interactions with O3 at subsite +1 and O2 at subsite −1 of the carbohydrate, respectively. The arrangement of O2, HO2, and H1 at subsite −1 seems to hinder the nucleophilic attack by water and the subsequent proton transfer from nucleophile to the base. In the trial structure from method (ii), Glu87 HE1 is located close to the O4 at subsite −1 (about 2.5Å away) making it favorable for proton transfer to the leaving group while Asp255 and Tyr351 were almost in the same positions as in the structure derived from method (i). The QM/MM calculations using the two trial systems generated with methods (i) and (ii) did not result in any nucleophilic attack at the anomeric center, due to the improper reactant state structures. In contrast, the reactant state structure obtained from method (iii) *i.e.*, the reverse reaction mechanism, in which the nucleophile (water molecule) was one of the reaction products, was energetically reasonable. The hydrolysis process (denoted as the forward reaction mechanism (Fig 3) in this article) follows from this state. The reaction pathways are same in both directions. Here, all the results are described along the forward reaction pathway, with the acid and base referred to accordingly.

### Molecular Dynamics (MD) Simulation

To examine the stability of the modeled product (CelS in complex with celloheptaose and cellobiose) structure, a MD simulation of the enzyme complex was carried out at 300K. The energy-minimum pathway is temperature-independent and thus also valid at the temperature optimum of the enzyme of 350K. For this simulation, the missing residues in both the enzyme and carbohydrate (missing subsite −1) were generated using the CHARMM [48] program. The topology and force field parameters for both the carbohydrate and enzyme were assigned from the CHARMM32 [48] parameter set. The protonation state and pKa of ionizable groups were calculated using the Poisson-Boltzman method for electrostatic calculations. In the product state, the catalytic acid (Glu87) was found to be deprotonated and the residues Tyr351 and Asp255 were protonated. The system was solvated in an equilibrated TIP3P [49] water box of size 84.0
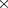
75.0
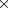
67.0 Å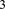. Solvent molecules within 2 Å from any protein heavy atom were deleted. The total number of atoms in the hydrated system was 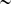
43000. Periodic boundary conditions were applied to the system in the canonical ensemble using the NAMD 2.6 package [50]. The velocity Verlet algorithm [51] was used to integrate the equations of motion with a time step of 1 fs. To neutralize the total charge of the system, 16 Na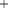 ions were added near the solvent-exposed surface of the protein. Electrostatic interactions were evaluated using the Particle Mesh Ewald formalism [52] as implemented in NAMD. The system was equilibrated for 1 ns followed by 7 ns of production run. The coordinates and velocities were stored every 60 fs.

### QM/MM Calculations

As a starting point for the QM/MM [53] calculations, an initial model of the enzyme-substrate complex was selected from the 300K MD-equilibrated structures and energy minimized with QM/MM. The QM/MM simulations were carried out using the Self-Consistent Charge Density Functional Tight Binding method (SCC-DFTB) [54] as implemented in CHARMM. The SCC-DFTB method is a fast semi-empirical density functional approach that has been extensively tested and applied to several enzymes [55]–[60] as well as a large number of model reactions of small organic molecules [61], [62]. These studies have shown that the DFTB method performs satisfactorily for the reactions involving the functional groups in this work.

**Figure 10:**
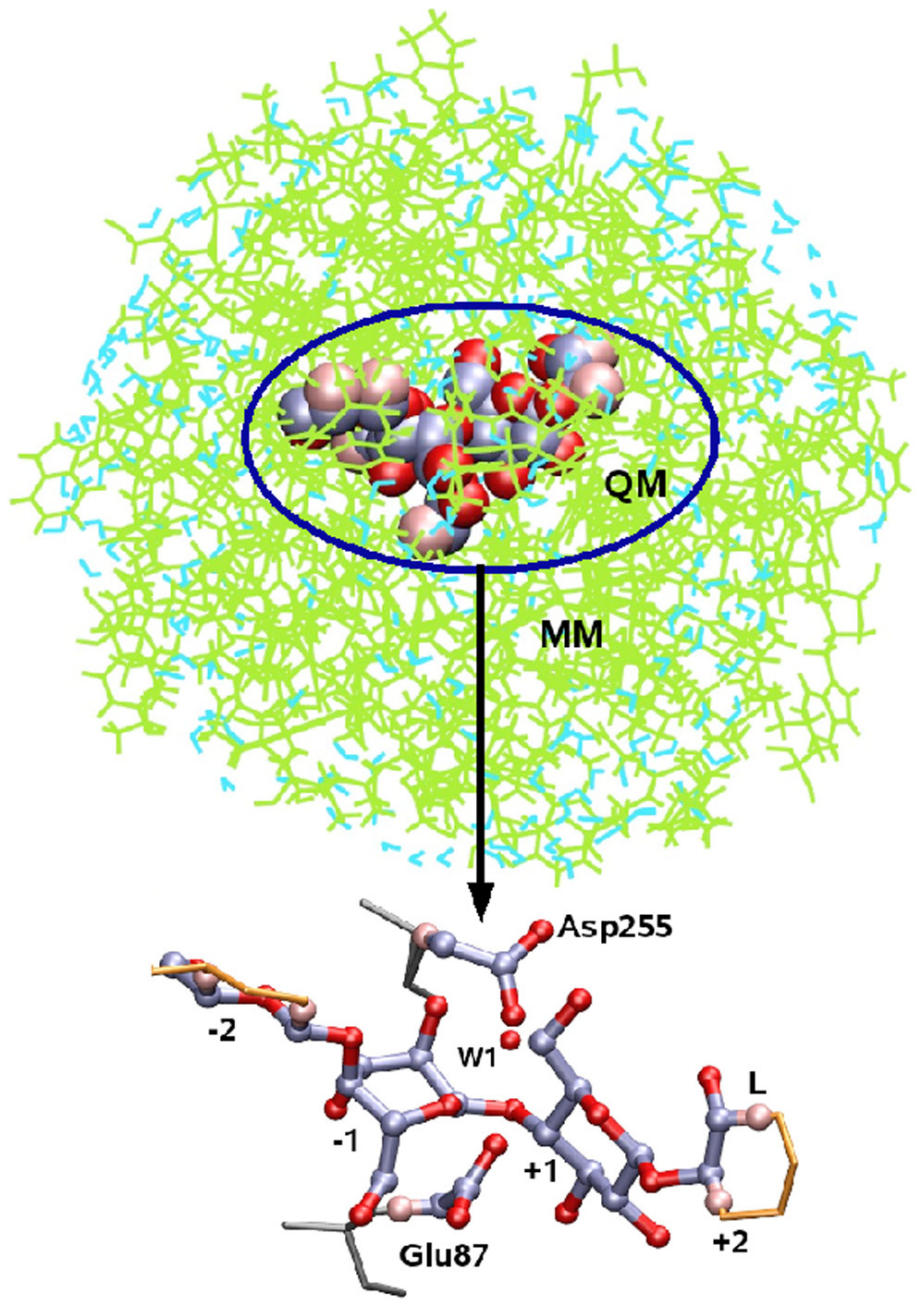
Setup for enzyme reaction in CelS with QM/MM method. QM region (VDW representation) consists of catalytic residues (Asp255 and Glu87), nucleophilic water (W1), and active part of substrate (subsites −1, and +1), while rest of enzyme (green), substrate (orange), and water (cyan) are in MM region. Inset shows only QM region and hydrogen link atoms (pink) used as boundary between MM and QM.

The QM region, comprising the sugar unit at subsite +1, Asp255, Glu87, and the chemically active part of the substrate (subsite −1) , is shown in Fig 10. Hydrogen link atoms [63] were placed between C1, C2 and C4, C5 at subsite −2, between C4, C5 and C3, C2 at subsite +2 on the carbohydrate, between C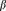, and C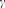 on Glu87 and between C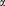, and C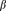 on Asp255. The atom names are given in Fig 3. The QM region consists of 78 atoms. All remaining atoms of the protein, carbohydrate, and solvent were treated using MM with the CHARMM force field. The substrate-enzyme complex was solvated in a 22Å sphere of TIP3P water [64] keeping the C1 atom of subsite −1 at the center. The stochastic boundary (SB) method [65] was used to represent environmental effects.
